# Population Prevalence of Deleterious *SGCE* Variants

**DOI:** 10.5334/tohm.567

**Published:** 2020-11-04

**Authors:** Mark S. LeDoux

**Affiliations:** 1 Veracity Neuroscience LLC, Memphis, Tennessee, US; 2 University of Memphis, Memphis, Tennessee, US

**Keywords:** *SGCE*, myoclonus, dystonia, missense pathogenic variant, splice-site pathogenic variant, stop-gained pathogenic variant

## Abstract

**Background:**

Myoclonus-Dystonia (M-D) is a pleiotropic neuropsychiatric disorder of variable penetrance. Pathogenic variants in *SGCE*, a maternally imprinted gene, are the most frequent known genetic cause of M-D. The population prevalence of *SGCE*-linked M-D is unknown, the pathogenicity of *SGCE* variants identified in patients with M-D may be indeterminant, and *SGCE* variants predicted to be deleterious by *in silico* analysis may appear in patients undergoing whole-exome or whole-genome sequencing for seemingly unrelated disorders. The Genome Aggregation Database (gnomAD) v2 provides variant data on 125,748 exomes and 15,708 genomes from unrelated individuals sequenced as part of various disease-specific and population genetic studies.

**Methods:**

*SGCE* variants included in the gnomAD v2 dataset were analyzed with Combined Annotation Dependent Depletion (CADD), and database for nonsynonymous single nucleotide polymorphisms’ functional predictions (dbNSFP). We determined the frequency of annotated *SGCE* variants, ranked by scores of deleteriousness, within the gnomAD v2 dataset. Deleteriousness scores were compared to a subset of published disease associated *SGCE* pathogenic variants.

**Results:**

Within gnomAD v2, there were 56, 408, and 1250 alleles harboring *SGCE* variants with CADD scores greater than 30, 25, and 20, respectively. We estimate that approximately 1/348 individuals in the United States population harbors an *SGCE* variant with a CADD score ≥ 25.

**Discussion:**

*SGCE* M-D may be underdiagnosed due to pleiotropy, mild phenotypes, variable penetrance, and impaired access to genetic testing. Due to the high population prevalence of deleterious *SGCE* variants, caution should be used when asserting pathogenicity without co-segregation analyses and expert neurological examination of phenotypes within pedigrees.

**Highlights:**

*In silico* analyses of a large population database of genetic variants revealed that over 0.2% of individuals in the United States harbor a highly deleterious *SGCE* variant. This finding suggests that M-D and minor phenotypic variants such as mild isolated myoclonus may be underdiagnosed.

## Introduction

Pathogenic variants in *SGCE* (Chr 7q21.3), a maternally imprinted gene, have been causally linked to Myoclonus-Dystonia (M-D) which is most commonly characterized by childhood onset of myoclonus and dystonia [1]. Dystonia can be generalized but typically affects the arm and neck. In some patients, the dystonia is mild to entirely absent. Myoclonus can also be generalized, but characteristically affects the arms and trunk. Psychiatric co-morbidities including depression, anxiety, bipolar disorder, phobias, alcoholism, and obsessive-compulsive disorder are part of the disease spectrum [2,3]. These psychiatric manifestations may occur in isolation in some members of affected pedigrees. Furthermore, the neuropsychiatric spectrum of *SGCE* pathogenic variants is broad and may include early gait dysfunction, isolated myoclonus, generalized dystonia, and cognitive impairment. Penetrance is incomplete and notable infrafamilial and extrafamilial phenotypic variability is well established [4]. Early and correct diagnosis may facilitate appropriate pharmacological and surgical interventions, and genetic counseling.

Little is known about the epidemiology of M-D. However, it is known that M-D affects most, if not all, racial groups including Chinese, Europeans, and Indians [5,6,7,8,9,10,11]. In general, most movement disorders experts would support the notion that the incidence of M-D is less than that of isolated cervical dystonia which has been reported as 0.80/100,000 person-years in Northern California [12]. In predominately European populations, prevalence estimates of cervical dystonia range from 28 to 183 cases/million [13]. Although commonly associated with childhood-onset generalized dystonia, DYT1 due to the classic ΔGAG deletion in *TOR1A*, may manifest as an M-D-like syndrome [14]. Based on analysis of 135,000 exomes, carrier prevalence of the ΔGAG deletion in *TOR1A* has been estimated at 176 to 261/million in the United States [15]. These epidemiological data from the most common forms of adult-onset focal dystonia and childhood-onset generalized dystonia provide reference points for interpretation of the *SGCE* M-D data reported herein.

## Methods

All *SGCE* variants (short and structural) reported in the genome Aggregation Database (gnomAD) v2 were included in my analysis. The v2 short variant data set includes 125,748 exomes and 15,708 genomes from unrelated subjects sequenced as part of various disease-specific and population genetic studies, totaling 141,456 subjects, and is aligned against the GRCh37/hg19 reference genome. The v2 release (gnomAD v2) comprises a total of 16 million single nucleotide variants (SNVs) and 1.2 million indels from 125,748 exomes. In gnomAD, all genomic rearrangements involving at least 50bp of DNA are defined as structural variants. Subjects known to be affected by severe pediatric disease, as well as their first-degree relatives, are not included in this dataset. However, some adult subjects with severe disease may still be included in the datasets, but likely at a frequency equivalent to or lower than that seen in the general population. In addition, gnomAD v2 parcels out a filtered non-neuro subset comprised of 104,068 exomes and 10,636 genomes.

I did not include the gnomAD v3 dataset for several reasons. The gnomAD v3 data set contains 71,702 whole genomes (and no exomes), all mapped to a different reference sequence (GRCh38/hg38). The gnomAD v2 and v3 datasets are not independent since most of the genomes from v2 are included in v3. At present, gnomAD v3 does not include a structural variant dataset. Variants were grouped based on gnomAD annotations (stop, frameshift, splice, missense, synonymous, intronic, start lost, 5’untranslated region [UTR], 3’UTR, and in-frame insertion) for downstream analyses. I did not include dubious variants flagged and filtered by gnomAD. Flagged variants did not pass the gnomAD quality control process and include those in low complexity regions, variants predicted to disrupt splicing outside the canonical splice site, and multi-nucleotide variants found in phase with another variant.

Variants reported by gnomAD were analyzed with Combined Annotation Dependent Depletion (CADD). CADD integrates multiple annotations by contrasting variants that survived natural selection with simulated pathogenic variants [16,17]. For the analyses reported here, I focused on CADD “PHRED-scaled” scores which represent the rank in order of magnitude terms rather than the precise rank itself. Reference genome single nucleotide variants at the 10% of CADD scores are assigned to CADD PHRED-10, top 1% to CADD PHRED-20, top 0.1% to CADD PHRED-30, etc. For *SGCE* variants, CADD raw scores ranged from –0.812 to 7.119, and CADD PHRED scores ranged from 0.008 to 38. CADD raw and PHRED scores were highly correlated (*r* = 0.975).

In addition to CADD, dbNSFP [18] was used for functional prediction and annotation of non-synonymous single-nucleotide variants (nsSNVs). Its current version (dbNSFP v4.0) is based on the Gencode release 29/Ensembl version 94 and includes a total of 84,013,490 nsSNVs and ssSNVs (splicing-site SNVs). dbNSFP compiles prediction scores from 29 algorithms (SIFT, Polyphen2-HDIV, MutationTaster2, MutationAssessor, FATHMM, MetaSVM, MetaLR, CADD, REVEL, PrimateAI, etc.), conservation scores, and includes other related information including allele frequencies observed in the 1000 Genomes Project phase 3 data, UK10K cohorts data, ExAC consortium data, gnomAD data and the NHLBI Exome Sequencing Project ESP6500 data, various gene IDs from different databases, functional descriptions of genes, gene expression and gene interaction information. Rare Exome Variant Ensemble Learner (REVEL) [19] and MetaLR [20] were used to predict the pathogenicity of nsSNVs. In comparison to most other prediction algorithms, REVEL and MetaLR show high overall performance and areas under the receiver operating characteristic curves [19].

The Human Gene Mutation Database® (HGMD) was used for identification of published *SGCE* variants reported in singletons and pedigrees with M-D. For comparison with population variants reported in gnomAD, I selected a subset of well-documented variants from multiple classes (missense, nonsense, splice, small deletions, and small insertions) included in 4 independent publications. Data from these 4 publications was reviewed to verify HGMD reporting. Of note, HGMD does not include all published *SGCE* variants and no attempt was made to scour the entire published literature to analyze all known disease-associated variants in *SGCE*.

## Results

### Analysis of published variants

Using the HGMD, I identified 25 disease-associated *SGCE* variants reported in 4 independent publications [4,21,22,23]. M-D has been linked to virtually all types of variants including interstitial deletions, single-exonic deletions, indels, in-frame deletions, non-synonymous single-nucleotide missense, and splice. The vast majority of indels lead to frameshifts and stops (Table 1), likely resulting in nonsense-mediated decay [24]. Two (c.812G>A, and c.289C>T) of the 25 selected variants are also present in gnomAD, presumably in two single individuals (Table 1). CADD_PHRED scores ranged from 21.2 for an in-frame deletion to 41 for two nonsense variants.

**Table 1:** *In silico* analysis of published *SGCE* MDS-linked variants.

Transcript Description	Annotation	Protein Description	Genomic Description	gnomAD Alleles	CADD PHRED	REVEL rankscore	MetaLR rankscore	Reference

NM_001099401.1:c.966delT	indel/frameshift/stop	NP_001092871.1:p.(Val323CysfsTer11)	GRCh37:7:94230028:CA:C	0	29.8	NA	NA	Schule et al. 2004 [21]
NM_001099401.1:c.179A>G	missense	NP_001092871.1:p.(His60Arg)	GRCh37:7:94259084:T:C	0	25.4	0.979	0.983	Schule et al. 2004 [21]
NM_001099401.1:c.232+2T>C	splice	NP_001092871.1:p.?	GRCh37:7:94259029:A:G	0	33	NA	NA	Du Montcel et al. 2006 [22]
NM_001099401.1: c.233-1G>T	splice	NP_001092871.1:p.?	GRCh37:7:94257672:C:A	0	35	NA	NA	Du Montcel et al. 2006 [22]
NM_001099401.1: c.232+1G>A	splice	NP_001092871.1:p.?	GRCh37:7:94259030:C:T	0	33	NA	NA	Du Montcel et al. 2006 [22]
NM_001099401.1: c.1114C>T	nonsense/stop	NP_001092871.1:p.(Arg372Ter)	GRCh37:7:94228226:G:A	0	41	NA	NA	Du Montcel et al. 2006 [22]
NM_001099401.1:c.300G>A	nonsense/stop	NP_001092871.1:p.(Trp100Ter)	GRCh37:7:94257604:C:T	0	37	NA	NA	Du Montcel et al. 2006 [22]
NM_001099401.1:c.208G>T	nonsense/stop	NP_001092871.1:p.(Glu70Ter)	GRCh37:7:94259055:C:A	0	40	NA	NA	Du Montcel et al. 2006 [22]
NM_001099401.1:c.812G>A	missense	NP_001092871.1:p.(Cys271Tyr)	GRCh37:7:94232615:C:T	1	35	0.986	0.994	Du Montcel et al. 2006 [22]
NM_001099401.1:c.344A>G	missense	NP_001092871.1:p.(Tyr115Cys)	GRCh37:7:94257560:T:C	0	27.8	0.997	0.992	Du Montcel et al. 2006 [22]
NM_001099401.1:c.742_745dup	indel/frameshift/stop	NP_001092871.1:p.(Ser249MetfsTer2)	GRCh37:7:94232681:C:CTACA	0	33	NA	NA	Du Montcel et al. 2006 [22]
NM_001099401.1:c.835_839del	indel/frameshift/stop	NP_001092871.1:p.(Thr279AlafsTer17)	GRCh37:7:94230155:CTTTGT:C	0	32	NA	NA	Du Montcel et al. 2006 [22]
NM_001099401.1:c.444_447del	indel/stop	NP_001092871.1:p.(Asn149Ter)	GRCh37:7:94252652:TATTA:T	0	33	NA	NA	Du Montcel et al. 2006 [22]
NM_001099401.1:c.107C>G	missense	NP_001092871.1:p.(Thr36Arg)	GRCh37:7:94285304:G:C	0	24	0.852	0.962	Raymond et al. 2008 [4]
NM_001099401.1:c.551T>C	missense	NP_001092871.1:p.(Leu184Pro)	GRCh37:7:94248181:A:G	0	23.8	0.987	0.995	Raymond et al. 2008 [4]
NM_001099401.1:c.662G>A	missense	NP_001092871.1:p.(Gly221Asp)	GRCh37:7:94248070:C:T	0	34	0.987	0.995	Peall et al. 2014 [23]
NM_001099401.1:c.109+5G>C	splice	NP_001092871.1:p.?	GRCh37:7:94285297:C:G	0	23.9	NA	NA	Peall et al. 2014 [23]
NM_001099401.1:c.289C>T	nonsense/stop	NP_001092871.1:p.(Arg97Ter)	GRCh37:7:94257615:G:A	1	29.8	NA	NA	Peall et al. 2014 [23]
NM_001099401.1:c.463+1G>A	splice	NP_001092871.1:p.?	GRCh37:7:94252636:C:T	0	35	NA	NA	Peall et al. 2014 [23]
NM_001099401.1:c.630_658del	indel/frameshift/stop	NP_001092871.1:p.(Val211GlyfsTer20)	GRCh37:7:94248073:TCCTTCAGGTCATTAATGGGAAGTGGCACC:T	0	33	NA	NA	Peall et al. 2014 [23]
NM_001099401.1:c.765_773del	indel/in-frame	NP_001092871.1:p.(Ile256_Cys258del)	GRCh37:7:94232653:ACATGTTATT:A	0	21.2	NA	NA	Peall et al. 2014 [23]
NM_001099401.1:c.771_772del	indel/stop	NP_001092871.1:p.(Cys258Ter)	GRCh37:7:94232654:CAT:C	0	32	NA	NA	Peall et al. 2014 [23]
NM_001099401.1:c.835_839del	indel/frameshift/stop	NP_001092871.1:p.(Thr279AlafsTer17)	GRCh37:7:94230155:CTTTGT:C	0	32	NA	NA	Peall et al. 2014 [23]
NM_001099401.1:c.1037+5G>A	splice	NP_001092871.1:p.?	GRCh37:7:94229953:C:T	0	25.7	NA	NA	Peall et al. 2014 [23]
NM_001099401.1:c.1114C>T	nonsense/stop	NP_001092871.1:p.(Arg372Ter)	GRCh37:7:94228226:G:A	0	41	NA	NA	Peall et al. 2014 [23]

Six missense variants are included in Table 1. CADD_PHRED scores range from 23.8 to 35. All 6 of these missense variants are predicted to be disease causing by MetaLR, MetaSVM, and MutationTaster. REVEL_rankscores ranged from 0.852 (p.Thr36Arg) to 0.997 (p.Tyr115Cys). However, the p.Thr36Arg variant was classified as T (tolerated) by PrimateAI_pred [25], and B (benign) by Polyphen_2_HDIV_pred. The male subject harboring the p.Thr36Arg variant had alcohol-responsive myoclonus but no dystonia or family history of M-D [4].

## *SGCE* variants in gnomAD v2

Recognizing that some fraction of the 282,646 *SGCE* alleles could contain two or more variants, a maximum of 134,145 alleles within the gnomAD database harbored a short *SGCE* variant (Table 2). As reported in gnomAD, *SGCE* has 7 polymorphisms (minor allele frequency > 5%). One of these is a nonsynonymous variant (p.Ser434Arg). Two others are found in low complexity regions of *SGCE* (Supplemental Table 1). Four structural variants, all intronic insertions, ranging in size from 243 bp to 322 bp, are present in 34 subjects within the gnomAD v2 dataset. The potential effects of these variants on gene expression and splicing are not known.

**Table 2:** GnomAD v2 *SGCE* variants.

CADD	# Variants	Total alleles*	Total individuals**	Population ratio	US population 330,270,291***	US cases corrected for imprinting

≥30	23 variants	56	56	1/2454	130,851	65,426
≥25	92 variants	408	406	1/348	948,817	474,409
≥20	265 variants	1,250	1,243	1/114	2,904,876	1,452,438
>0	780 variants	134,145	113,162	1/1.25	264,458,345	1,3222,917

* max: 282,646. ** correction for homozygotes. *** Based on US Census Bureau estimates.

Of the 780 short variants reported in gnomAD v2, 265 had CADD scores ≥ 20. This subset of variants was present in a maximum of 1250 alleles. Based on data contained within Table 1, a more restrictive group of 92 more deleterious and possibly pathogenic variants with CADD_PHRED scores ≥25 was found in 406 individuals. Extrapolating to the current population in the United States (US Census Bureau, www.census.gov), nearly one million individuals in the US (1/348 or 0.287%) harbors a highly deleterious and possibly pathogenic *SGCE* variant in their genome. Limiting analysis to the most deleterious variants, and, after correction for imprinting, there could be an estimated 65,426 cases of M-D in the US.

CADD_PHRED scores were also generated for the 720 *SGCE* variants present in the gnomAD non-neuro v2 dataset derived from 104,068 exomes and 10,636 genomes. A total of 254 variants had CADD scores ≥ 20: 22 ≥ 30, 67 ≥ 25 and < 30, and 162 ≥ 20 and < 25. Three variants were homozygous. The total number of variants and their CADD scores are proportional to the data derived from the entirety of gnomAD v2. Therefore, deleterious *SGCE* variants are not concentrated in “neuro” exomes and genomes within gnomAD v2.

## Variant annotation

As gleaned from Tables 1 and 3, nonsense, frameshift and splice pathogenic variants leading to NMD or truncated proteins are associated with the highest CADD scores of deleteriousness. Of the 23 variants with CADD_PHRED scores ≥30, 3 were stops, 4 were frameshifts leading to premature termination, 4 were located within canonical splice sites, and 12 were missense (Table 3). In contrast, a much broader array of variants had CADD_PHRED scores between 20 and 25. In particular, among 173 variants in this grouping, 133 were missense, 7 were synonymous and 15 were in the 5’UTR. In contrast, there was only 1 stop or frameshift variant with a CADD score between 20 and 25, supporting the notion that more deleterious variants are poorly tolerated and may be causally associated with neurological manifestations. It should be noted that synonymous and 5’UTR variants are important causes of disease. Synonymous variants can alter mRNA stability and splicing and the rate of translation. Similarly, variants in the 5’UTR can exert deleterious effects on translation.

**Table 3:** Variant annotation and CADD scores (gnomAD v2).

	CADD ≥30		CADD ≥25 & <30		CADD ≥20 & <25	

Annotation	Number of variants	Total alleles	Number of variants	Total alleles	Number of variants	Total alleles

Stop	3	3	2	24	0	0
Frameshift	4	4	0	0	1	1
Splice	4	14	1	1	4	6
Missense	12	35	66	327	133	743
Synonymous	0	0	0	0	7	30
Intronic	0	0	0	0	9	20
Start lost	0	0	0	0	1	1
5’UTR	0	0	0	0	15	32
3’UTR	0	0	0	0	0	0
In-Frame Insertion/Deletion	0	0	0	0	3	9
All variants	23	56	69	352	173	842

## Missense variants

A maximum of 1216 gnomAD v2 alleles harbored a missense variant predicted to be disease-causing by MetaLR with a reliability_Index of 9 or 10 (Table 4). For this group of variants, MetaLR_rankscores ranged from 0.99 to 0.95 with a median value of 0.97. For comparative purposes, I compared and correlated CADD_PHRED scores with MetaLR-rankscores and REVEL_rankscores (Supplemental Table 2). To apply a threshold value for deleteriousness, it is generally recommended that a CADD-PHRED score of 15 is chosen for the process of identifying potentially pathogenic variants (cadd.gs.washington.edu/info). This value is near the low CADD_PHRED score of 14.45. Given that correlations among CADD-PHRED scores, MetaLR_rankscores and REVEL_rankscores were only moderate (r > 0.5), reliance on a single measure of deleteriousness could lead to missed assignments of pathogenicity (Supplemental Table 2).

**Table 4:** MetaLR, REVEL and CADD scores for 237 missense variants.

	High Score	Low Score	Median Score

MetaLR_rankscore*	0.99	0.95	0.97
REVEL_rankscore	0.99	0.54	0.87
CADD_PHRED	34.00	14.45	23.20

* MetaLR (D-disease causing, Reliability_index of 9 or 10).

## Discussion

My analyses of the gnomAD database indicate that deleterious variants in *SGCE* are common in presumably normal populations. This finding should inform interpretation of whole-exome sequencing (WES) and whole-genome sequencing (WGS) performed on individuals and populations for other purposes. There are several interpretations of my findings. First, *in silico* analyses of deleteriousness may be weak predictors of pathogenicity, particularly for *SGCE* and M-D. Second, individuals with deleterious *SGCE* variants may have very mild, unrecognized, clinical manifestations or isolated psychiatric disease. Third, M-D may be misdiagnosed or underdiagnosed, and, in this regard, most subjects included in the gnomAD database did not undergo neurological examination by an expert in movement disorders.

Additional limitations of my work should be highlighted. First, WES and WGS are associated with small false positive and false negative error rates. Although uncommon, M-D can be cause by large interstitial deletions which are often missed by short-read next-generation sequencing. These large structural variants would increase the predicted number of cases in the population. The single nucleotide and indel variants included in gnomAD were not confirmed with bidirectional Sanger sequencing. As such, it is possible that a small percentage of the reported variants were short-read errors. Caution should be used with examination of the gnomAD database and most other large genomic/genetic databases. Ideally, a neurological control genetic database should be restricted to neurologically- and psychiatrically-normal adults with no first- or second-degree relatives with neurological or psychiatric disease. The limitations of entirely *in silico* approaches are well established and reliable, inexpensive, high-throughput functional assays for *SGCE* variants are not available. Rather than simple reliance on CADD, my analyses suggest that clinical geneticists should use multiple *in silico* tools and query population control databases when evaluating the potential pathogenicity of *SGCE* variants, particularly missense variants.

Using a minimum ΔGAG carrier prevalence of 176/million and penetrance of 35%, an estimated 62 cases of DYT1 dystonia/million are present in the United States [15]. However, the actual number of DYT1 cases seen by movement disorders experts in the United States would seem much lower. This suggests that population penetrance may be considerably less than the penetrance within individual families, perhaps driven by other variants in *cis* or *trans*. In the context of M-D, our data predicts a population prevalence of 198 cases/million which is somewhat higher than the maximum estimated prevalence of cervical dystonia in the United States [13]. True penetrance can only be determined by expert examination of carriers within a population and variants databases like gnomAD are obviously limited in this regard.

*SGCE*-associated neuropsychiatric disease may be underrecognized by pediatric neurologists and psychiatrists. Neurological manifestations can range from early mild gait dysfunction or subtle myoclonus to generalized dystonia with cognitive dysfunction. M-D may be misdiagnosed as Tourette syndrome, myoclonic epilepsy, dyskinetic cerebral palsy, or isolated cervical dystonia with associated appendicular tremor [26]. Consideration of a genetic etiology may be dismissed due to maternal imprinting and broad phenotypic variability in individual pedigrees. The positive effects of alcohol on motor manifestations are rarely identified in children. Timely diagnosis of *SGCE*-associated neuropsychiatric disease is important for individual patients and pedigrees given that effective treatments are available for myoclonus, dystonia, anxiety, depression, and other disease manifestations.

Given the phenotypic variability of dystonia and other disorders of the motor system along with the declining costs of next-generation sequencing (NGS), multi-gene panels, WES and WGS are being increasing utilized for genetic diagnoses. For instance, Invitae (www.invitae.com) offers a comprehensive dystonia panel that includes *SGCE* and 17 other genes. Their panel is sequenced to high depth (50x minimum) to detect SNVs, indels, exon-level deletions/duplications, and large copy number variants. Among 1,910 patients with a clinical diagnosis of dystonia included in a recent report, 7.9% were given a molecular diagnosis and 11.8% were found to have a variant of unknown significance [27]. The genes with highest yield were *SGCE* (20.5%) and *TOR1A* (19.9%) [27]. For comparison, within the ClinVar database on July 3, 2020, there are 215 accessions associated with *SGCE*, but only 122 associated with *TOR1A*. These evidences suggest that *SGCE*-associated dystonia is perhaps more common in clinics than previously recognized.

In conclusion, we have shown that *SGCE* variants predicted to be highly deleterious are common in population and non-neurological disease controls. Accordingly, *SGCE*-associated neuropsychiatric disease may be underrecognized by clinicians. Alternatively, the population penetrance of deleterious variants in *SGCE* may be quite low. Ideally, expert examination of pedigrees and co-segregation should be used to establish the causality of *SGCE* variants identified by routine Sanger sequencing, next-generation multi-gene panels, WES or WGS. Future work should focus on environmental and genetic contributions to penetrance.

## Additional File

The additional file for this article can be found as follows:

10.5334/tohm.567.s1 Supplementary Data. Supplemental Tables 1 and 2.

## References

[1] Zimprich A, Grabowski M, Asmus F, Naumann M, Berg D, Bertram M, et al. Mutations in the gene encoding epsilon-sarcoglycan cause myoclonus-dystonia syndrome. Nat Genet. 2001; 29(1): 66–9. DOI: 10.1038/ng709 11528394

[2] Timmers ER, Smit M, Kuiper A, Bartels AL, van der Veen S, van der Stouwe AMM, et al. Myoclonus-dystonia: Distinctive motor and non-motor phenotype from other dystonia syndromes. Parkinsonism & related disorders. 2019; 69: 85–90. DOI: 10.1016/j.parkreldis.2019.10.015 31706131

[3] Peall KJ, Dijk JM, Saunders-Pullman R, Dreissen YE, van Loon I, Cath D, et al. Psychiatric disorders, myoclonus dystonia and SGCE: an international study. Ann Clin Transl Neurol. 2016; 3(1): 4–11. DOI: 10.1002/acn3.263 26783545 PMC4704478

[4] Raymond D, Saunders-Pullman R, de Carvalho Aguiar P, Schule B, Kock N, Friedman J, et al. Phenotypic spectrum and sex effects in eleven myoclonus-dystonia families with epsilon-sarcoglycan mutations. Movement disorders: official journal of the Movement Disorder Society. 2008; 23(4): 588–92. DOI: 10.1002/mds.21785 18175340

[5] Chen XP, Zhang YW, Zhang SS, Chen Q, Burgunder JM, Wu SH, et al. A novel mutation of the epsilon-sarcoglycan gene in a Chinese family with myoclonus-dystonia syndrome. Movement disorders: official journal of the Movement Disorder Society. 2008; 23(10): 1472–5. DOI: 10.1002/mds.22008 18581468

[6] Borges V, Aguiar Pde C, Ferraz HB, Ozelius LJ. Novel and de novo mutations of the SGCE gene in Brazilian patients with myoclonus-dystonia. Movement disorders: official journal of the Movement Disorder Society. 2007; 22(8): 1208–9. DOI: 10.1002/mds.21380 17394244

[7] Rachad L, El Otmani H, Karkar A, El Moutawakil B, El Kadmiri N, Nadifi S. Screening for SGCE mutations in Moroccan sporadic patients with Myoclonus-Dystonia syndrome. Neuroscience letters. 2019; 703: 1–4. DOI: 10.1016/j.neulet.2019.03.003 30849405

[8] Jain P, Sharma S, van Ruissen F, Aneja S. Myoclonus-dystonia: An under-recognized entity – Report of 5 cases. Neurol India. 2016; 64(5): 980–3. DOI: 10.4103/0028-3886.190255 27625242

[9] Thummler S, Giuliano F, Pincemaille O, Saugier-Veber P, Perelman S. Myoclonus in fraternal twin toddlers: a French family with a novel mutation in the SGCE gene. Eur J Paediatr Neurol. 2009; 13(6): 559–61. DOI: 10.1016/j.ejpn.2008.11.009 19147379

[10] Nardocci N. Myoclonus-dystonia syndrome. Handb Clin Neurol. 2011; 100: 563–75. DOI: 10.1016/B978-0-444-52014-2.00041-0 21496608

[11] Chung EJ, Lee WY, Kim JY, Kim JH, Kim GM, Ki CS, et al. Novel SGCE gene mutation in a Korean patient with myoclonus-dystonia with unique phenotype mimicking Moya-Moya disease. Movement disorders: official journal of the Movement Disorder Society. 2007; 22(8): 1206–7. DOI: 10.1002/mds.21093 17394247

[12] Marras C, Van den Eeden SK, Fross RD, Benedict-Albers KS, Klingman J, Leimpeter AD, et al. Minimum incidence of primary cervical dystonia in a multiethnic health care population. Neurology. 2007; 69(7): 676–80. DOI: 10.1212/01.wnl.0000267425.51598.c9 17698789

[13] Defazio G, Jankovic J, Giel JL, Papapetropoulos S. Descriptive epidemiology of cervical dystonia. Tremor Other Hyperkinet Mov (N Y). 2013; 3 DOI: 10.5334/tohm.170 PMC3822401 24255801

[14] Gatto EM, Pardal MM, Micheli FE. Unusual phenotypic expression of the DYT1 mutation. Parkinsonism & related disorders. 2003; 9(5): 277–9. DOI: 10.1016/S1353-8020(02)00128-1 12781594

[15] Park J, Damrauer SM, Baras A, Reid JG, Overton JD, Gonzalez-Alegre P. Epidemiology of DYT1 dystonia: Estimating prevalence via genetic ascertainment. Neurology Genetics. 2019; 5(5): e358 DOI: 10.1212/NXG.0000000000000358 31583275 PMC6745720

[16] Kircher M, Witten DM, Jain P, O’Roak BJ, Cooper GM, Shendure J. A general framework for estimating the relative pathogenicity of human genetic variants. Nat Genet. 2014; 46(3): 310–5. DOI: 10.1038/ng.2892 24487276 PMC3992975

[17] Rentzsch P, Witten D, Cooper GM, Shendure J, Kircher M. CADD: predicting the deleteriousness of variants throughout the human genome. Nucleic acids research. 2019; 47(D1): D886–D94. DOI: 10.1093/nar/gky1016 30371827 PMC6323892

[18] Liu X, Wu C, Li C, Boerwinkle E. dbNSFP v3.0: A One-Stop Database of Functional Predictions and Annotations for Human Nonsynonymous and Splice-Site SNVs. Hum Mutat. 2016; 37(3): 235–41. DOI: 10.1002/humu.22932 26555599 PMC4752381

[19] Ioannidis NM, Rothstein JH, Pejaver V, Middha S, McDonnell SK, Baheti S, et al. REVEL: An Ensemble Method for Predicting the Pathogenicity of Rare Missense Variants. American journal of human genetics. 2016; 99(4): 877–85. DOI: 10.1016/j.ajhg.2016.08.016 27666373 PMC5065685

[20] Dong C, Wei P, Jian X, Gibbs R, Boerwinkle E, Wang K, et al. Comparison and integration of deleteriousness prediction methods for nonsynonymous SNVs in whole exome sequencing studies. Human molecular genetics. 2015; 24(8): 2125–37. DOI: 10.1093/hmg/ddu733 25552646 PMC4375422

[21] Schule B, Kock N, Svetel M, Dragasevic N, Hedrich K, De Carvalho Aguiar P, et al. Genetic heterogeneity in ten families with myoclonus-dystonia. Journal of neurology, neurosurgery, and psychiatry. 2004; 75(8): 1181–5. DOI: 10.1136/jnnp.2003.027177 PMC1739169 15258227

[22] Tezenas du Montcel S, Clot F, Vidailhet M, Roze E, Damier P, Jedynak CP, et al. Epsilon sarcoglycan mutations and phenotype in French patients with myoclonic syndromes. J Med Genet. 2006; 43(5): 394–400. DOI: 10.1136/jmg.2005.036780 16227522 PMC2564513

[23] Peall KJ, Kurian MA, Wardle M, Waite AJ, Hedderly T, Lin JP, et al. SGCE and myoclonus dystonia: motor characteristics, diagnostic criteria and clinical predictors of genotype. Journal of neurology. 2014; 261(12): 2296–304. DOI: 10.1007/s00415-014-7488-3 25209853 PMC4495322

[24] Xiao J, Nance MA, LeDoux MS. Incomplete nonsense-mediated decay facilitates detection of a multi-exonic deletion mutation in SGCE. Clinical genetics. 2013; 84(3): 276–80. DOI: 10.1111/cge.12059 23140253

[25] Sundaram L, Gao H, Padigepati SR, McRae JF, Li Y, Kosmicki JA, et al. Predicting the clinical impact of human mutation with deep neural networks. Nat Genet. 2018; 50(8): 1161–70. DOI: 10.1038/s41588-018-0167-z 30038395 PMC6237276

[26] Varga MG, Nand NP, LeDoux MS. Delayed diagnoses of SGCE myoclonus-dystonia. Tremor Other Hyperkinet Mov (N Y). 2020 DOI: 10.5334/tohm.334 PMC7394200 32775037

[27] Winder TL, Tan CA, Klemm S, White H, Westbrook JM, Wang JZ, et al. Clinical utility of multigene analysis in over 25,000 patients with neuromuscular disorders. Neurology Genetics. 2020; 6(2): e412 DOI: 10.1212/NXG.0000000000000412 32337338 PMC7164976

