# Supplementary material for: Population Prevalence of Deleterious SGCE Variants
Source: Tremor Other Hyperkinet Mov (N Y). 2020 Nov 4;10:50. doi: 10.5334/tohm.567 (PMC7646276; doi:10.5334/tohm.567)
Supplement: Supplementary Data. — Supplemental Tables 1 and 2. [file tohm-10-1-567-s1.pdf]

Supplemental Table 1

SGCE polymorphisms (minor allele frequency > 5%)

Transcript Consequence	Protein Consequence	Annotation	Flags	Allele Count	Allele Number	Allele Frequency	Homozygote Count
c.1294A>C	p.Ser432Arg	missense_variant	mnv	26403	34026	0.776	10427
c.390+63_390+64dupGT		intron_variant	lcr	10957	29308	0.374	1875
c.1372+811T>A		intron_variant		8214	31356	0.262	1568
c.109+11308T>C		intron_variant		26678	163184	0.163	2389
c.391-43A>C		intron_variant		39115	250176	0.156	3389
c.109+11270A>G		intron_variant		4027	31358	0.128	311
c.390+59_390+64dupGTGTGT		intron_variant	lcr	1658	29308	0.057	52

*mnv, multi-nucleotide variant found in phase with another variant in some individuals, altering the amino acid sequence.
**lcr, variant found in low complexity region – annotation or quality dubious.

Supplemental Table 2

Correlation matrix for *in silico* predictors of deleteriousness

	MetaLR_rankscore	REVEL_rankscore	CADD_PHRED
MetaLR_rankscore	1	0.629	0.504
REVEL_rankscore	0.629	1	0.528
CADD_PHRED	0.504	0.528	1
